# Supplementary material for: Association between SNPs in Leptin Pathway Genes and Anthropometric, Biochemical, and Dietary Markers Related to Obesity
Source: Genes (Basel). 2022 May 25;13(6):945. doi: 10.3390/genes13060945 (PMC9222344; doi:10.3390/genes13060945)
Supplement: Supplementary file 1 [file genes-13-00945-s001.zip › genes-1702404-supplementary.pdf]

Table S1. Determination of the influence of SNPs on classic marker of obesity.

SNP	Obesity marker	Model	Mean	S.D.	p-Value
		LEP			
rs10244329	Body Fat (%)	XX	24.39	10.51	0.045
		Xx + xx	26.73	9.07	
	Waist circumference	XX	77.80	13.29	0.011
		Xx + xx	81.52	11.81	
	BMI	XX	22.60	5.09	0.014
		Xx + xx	23.95	4.29	
rs11760956	Body Fat (%)	XX	25.05	9.52	0.011
		Xx + xx	27.15	9.11	
	Waist circumference	XX	79.23	11.77	0.009
		Xx + xx	81.97	12.14	
	BMI	XX	23.03	4.45	0.004
		Xx + xx	24.15	4.36	
rs4731426	Body Fat (%)	XX	25.93	8.82	0.465
		Xx + xx	26.59	9.46	
	Waist circumference	XX	81.02	11.64	0.995
		Xx + xx	81.02	12.24	
	BMI	XX	23.93	4.23	0.599
		Xx + xx	23.71	4.49	
		LEPR			
rs111573261	Body Fat (%)	XX	26.42	9.27	0.813
		Xx + xx	26.84	9.61	
	Waist circumference	XX	80.93	11.76	0.631
		Xx + xx	81.98	12.38	
	BMI	XX	23.76	4.35	0.913
		Xx + xx	23.67	4.17	
rs78862345	Body Fat (%)	XX	26.42	9.27	0.813
		Xx + xx	26.84	9.61	
	Waist circumference	XX	80.93	11.76	0.631
		Xx + xx	81.98	12.38	
	BMI	XX	23.76	4.35	0.913
		Xx + xx	23.67	4.17	
rs114280901	Body Fat (%)	XX	26.50	9.31	0.435
		Xx + xx	24.76	8.39	
	Waist circumference	XX	81.06	11.80	0.415
		Xx + xx	78.85	11.35	
	BMI	XX	23.80	4.34	0.174

		Xx + xx	22.42	4.17	
rs12035604	Body Fat (%)	XX	25.80	9.24	0.166
		Xx + xx	26.91	9.30	
	Waist circumference	XX	80.62	11.30	0.529
		Xx + xx	81.25	12.13	
	BMI	XX	23.44	4.01	0.148
		Xx + xx	23.97	4.55	
rs1137101	Body Fat (%)	XX	25.53	8.82	0.14
		Xx + xx	26.81	9.45	
	Waist circumference	XX	81.05	11.11	0.93
		Xx + xx	80.96	12.06	
	BMI	XX	23.51	3.87	0.374
		Xx + xx	23.85	4.51	
rs4655723	Body Fat (%)	XX	25.84	9.22	0.162
		Xx + xx	26.95	9.32	
	Waist circumference	XX	80.69	11.19	0.584
		Xx + xx	81.23	12.27	
	BMI	XX	23.50	4.04	0.186
		Xx + xx	23.97	4.57	
rs6700896	Body Fat (%)	XX	25.78	8.89	0.35
		Xx + xx	26.65	9.40	
	Waist circumference	XX	81.21	11.40	0.798
		Xx + xx	80.92	11.91	
	BMI	XX	23.65	4.25	0.743
		Xx + xx	23.78	4.37	
rs1805096	Body Fat (%)	XX	25.72	8.85	0.308
		Xx + xx	26.67	9.41	
	Waist circumference	XX	80.98	11.53	0.999
		Xx + xx	80.99	11.87	
	BMI	XX	23.62	4.26	0.695
		Xx + xx	23.79	4.36	
rs9436301	Body Fat (%)	XX	26.21	9.41	0.556
		Xx + xx	26.67	9.18	
	Waist circumference	XX	81.18	11.88	0.705
		Xx + xx	80.80	11.70	
	BMI	XX	23.76	4.24	0.945
		Xx + xx	23.74	4.43	
rs77451629	Body Fat (%)	XX	26.48	9.26	0.549
		Xx + xx	24.70	10.89	

	Waist circumference	XX	81.04	11.80	0.4
		Xx + xx	78.02	11.09	
	BMI	XX	23.78	4.34	0.224
		Xx + xx	22.17	3.93	
rs1171278	Body Fat (%)	XX	26.31	9.28	0.722
		Xx + xx	26.59	9.30	
	Waist circumference	XX	81.01	11.52	0.996
		Xx + xx	80.96	12.06	
	BMI	XX	23.72	4.17	0.88
		Xx + xx	23.78	4.50	
rs12145690	Body Fat (%)	XX	27.24	9.24	0.259
		Xx + xx	26.20	9.29	
	Waist circumference	XX	81.08	11.72	0.911
		Xx + xx	80.96	11.81	
	BMI	XX	23.93	4.50	0.591
		Xx + xx	23.70	4.29	
rs9436301	Body Fat (%)	XX	26.21	9.41	0.556
		Xx + xx	26.67	9.18	
	Waist circumference	XX	81.18	11.88	0.705
		Xx + xx	80.80	11.70	
	BMI	XX	23.76	4.24	0.945
		Xx + xx	23.74	4.43	
rs11208648	Body Fat (%)	XX	26.42	9.26	0.783
		Xx + xx	26.95	10.00	
	Waist circumference	XX	81.03	11.81	0.649
		Xx + xx	79.96	11.32	
	BMI	XX	23.77	4.33	0.665
		Xx + xx	23.39	4.57	
rs970467	Body Fat (%)	XX	26.40	9.33	0.882
		Xx + xx	26.53	9.20	
	Waist circumference	XX	80.98	11.79	0.982
		Xx + xx	81.00	11.81	
	BMI	XX	23.69	4.19	0.601
		Xx + xx	23.89	4.63	
rs10128072	Body Fat (%)	XX	26.29	9.24	0.653
		Xx + xx	26.64	9.35	
	Waist circumference	XX	81.11	11.99	0.78
		Xx + xx	80.83	11.54	
	BMI	XX	23.70	4.19	0.75

		Xx + xx	23.82	4.51	
rs1171278	Body Fat (%)	XX	26.31	9.28	0.722
		Xx + xx	26.59	9.30	
	Waist circumference	XX	81.01	11.52	0.996
		Xx + xx	80.96	12.06	
	BMI	XX	23.72	4.17	0.88
		Xx + xx	23.78	4.50	
rs77451629	Body Fat (%)	XX	26.48	9.26	0.549
		Xx + xx	24.70	10.89	
	Waist circumference	XX	81.04	11.80	0.392
		Xx + xx	78.02	11.09	
	BMI	XX	23.78	4.34	0.224
		Xx + xx	22.17	3.93	
rs2025804	Body Fat (%)	XX	26.45	8.58	0.996
		Xx + xx	26.44	9.41	
	Waist circumference	XX	82.24	12.81	0.292
		Xx + xx	80.77	11.60	
	BMI	XX	24.39	4.34	0.144
		Xx + xx	23.64	4.33	
rs2104564	Body Fat (%)	XX	27.10	8.62	0.513
		Xx + xx	26.34	9.38	
	Waist circumference	XX	81.94	12.24	0.451
		Xx + xx	80.84	11.72	
	BMI	XX	24.38	4.24	0.177
		Xx + xx	23.66	4.35	
rs1751492	Body Fat (%)	XX	26.39	8.20	0.956
		Xx + xx	26.45	9.42	
	Waist circumference	XX	80.37	11.75	0.657
		Xx + xx	81.06	11.79	
	BMI	XX	23.82	3.93	0.884
		Xx + xx	23.74	4.39	
rs11208659	Body Fat (%)	XX	26.46	9.22	0.903
		Xx + xx	26.32	9.74	
	Waist circumference	XX	81.12	11.93	0.48
		Xx + xx	80.11	10.80	
	BMI	XX	23.82	4.38	0.323
		Xx + xx	23.30	4.04	
rs1045895	Body Fat (%)	XX	27.08	8.98	0.11
		Xx + xx	25.82	9.54	

	Waist circumference	XX	81.50	11.97	0.301
		Xx + xx	80.48	11.60	
	BMI	XX	24.09	4.39	0.06
		Xx + xx	23.41	4.26	
rs9436748	Body Fat (%)	XX	26.92	9.03	0.253
		Xx + xx	26.02	9.50	
	Waist circumference	XX	81.40	12.01	0.428
		Xx + xx	80.61	11.58	
	BMI	XX	24.08	4.44	0.084
		Xx + xx	23.45	4.23	
rs10749753	Body Fat (%)	XX	26.73	9.03	0.403
		Xx + xx	26.07	9.61	
	Waist circumference	XX	81.40	11.91	0.338
		Xx + xx	80.45	11.61	
	BMI	XX	23.99	4.33	0.134
		Xx + xx	23.44	4.33	
rs72683113	Body Fat (%)	XX	26.53	9.20	0.561
		Xx + xx	25.80	9.98	
	Waist circumference	XX	81.07	11.73	0.651
		Xx + xx	80.33	12.26	
	BMI	XX	23.80	4.33	0.482
		Xx + xx	23.39	4.41	
rs1137101	Body Fat (%)	XX	25.53	8.82	0.14
		Xx + xx	26.81	9.45	
	Waist circumference	XX	81.05	11.11	0.93
		Xx + xx	80.96	12.06	
	BMI	XX	23.51	3.87	0.374
		Xx + xx	23.85	4.51	
		PSKC1			
rs17392686	Body Fat (%)	XX	26.46	9.35	0.913
		Xx + xx	26.92	8.10	
	Waist circumference	XX	81.04	12.09	0.898
		Xx + xx	81.73	11.73	
	BMI	XX	23.80	4.46	0.713
		Xx + xx	23.07	3.99	
rs271923	Body Fat (%)	XX	27.19	9.99	0.53
		Xx + xx	26.38	9.26	
	Waist circumference	XX	82.37	13.71	0.363
		Xx + xx	80.89	11.88	

	BMI	XX	24.05	4.48	0.642
		Xx + xx	23.77	4.46	
		MC4R			
rs34114122	Body Fat (%)	XX	26.43	9.31	0.83
		Xx + xx	25.89	8.39	
	Waist circumference	XX	81.04	11.91	0.237
		Xx + xx	77.48	10.34	
	BMI	XX	23.77	4.34	0.514
		Xx + xx	23.03	4.40	
rs2229616	Body Fat (%)	XX	26.45	9.29	0.489
		Xx + xx	23.81	8.21	
	Waist circumference	XX	81.03	11.90	0.127
		Xx + xx	74.13	7.32	
	BMI	XX	23.77	4.35	0.347
		Xx + xx	22.10	2.53	
		POMC			
rs28932472	Body Fat (%)	XX	25.72	9.01	0.138
		Xx + xx	26.91	9.45	
	Waist circumference	XX	80.78	10.96	0.742
		Xx + xx	81.10	12.51	
	BMI	XX	23.48	3.93	0.216
		Xx + xx	23.94	4.60	
